# Optimal Management of the Hepatitis B Patient Who Desires Pregnancy or Is Pregnant

**DOI:** 10.1007/s11901-012-0130-x

**Published:** 2012-05-26

**Authors:** Natalie H. Bzowej

**Affiliations:** California Pacific Medical Center, 2340 Clay Street, Suite 312, San Francisco, CA 94115 USA

**Keywords:** Hepatitis B, Pregnancy, Perinatal Transmission, Treatment, Antiviral, Nucleoside, Nucleotide, Lamivudine, Tenofovir, Telbivudine

## Abstract

Women of childbearing age with recognized hepatitis B infection should have their liver disease assessed before pregnancy occurs since the management of hepatitis B virus (HBV) infection in this setting is complex. Initiation of treatment in a woman of child-bearing age will depend on when she intends on conceiving, as well as the severity of her liver disease. During pregnancy, all decisions about initiating, continuing or stopping HBV therapy must include an analysis of the risks and benefits for both mother and fetus. The trimester of the pregnancy and the stage of the mother’s liver disease are important factors. Treatment in the third trimester may be considered to aid in prevention of perinatal transmission, which appears to be most pronounced in mothers with high viral loads. Consideration of initiation of third trimester treatment should occur after a high viral load is documented in the latter part of the second trimester, to allow adequate time for initiation of antiviral therapy with significant viral suppression before delivery. This discussion should include the topic of breastfeeding, since it is generally not recommended while on antiviral therapy. Until recently lamivudine and tenofovir appeared to be the therapeutic options with the most reasonable safety data in pregnancy. There are emerging data that telbivudine may also be considered in this setting.

## Introduction

It is estimated that 350–400 million individuals worldwide are chronically infected with HBV [1]. In regions with high prevalence, infection is most commonly acquired through either perinatal or horizontal transmission [2, 3]. The risk of progression to chronic HBV infection is inversely proportional to the age at which the infection was acquired. Without immunoprophylaxis, up to 90 % of infants born to hepatitis B e antigen (HBeAg)-positive mothers become infected. In contrast, only 20 % to 30 % of children exposed between age 1 and 5 years, and fewer than 5 % of adults become infected [4–6]. Thus, women of childbearing age with chronic HBV infection remain an important source for continued viral transmission.

This article will address a number of issues pertinent to hepatitis B therapy in pregnancy. First, the woman of childbearing age who may require therapy for hepatitis B with particular emphasis on the timing of therapy, choice of agent and the patient’s desire to conceive in the future will be discussed. Second, an approach to the woman who is newly diagnosed with hepatitis B early in pregnancy will be outlined. In addition, whether therapy should be continued, switched or stopped if an HBV-infected woman is on treatment and becomes pregnant will be debated. Finally, the question of whether a pregnant woman should be treated in the third trimester to help prevent perinatal transmission will be addressed in a proposed algorithm for the management of HBV in pregnancy.

## Therapy for HBV in Women of Childbearing Age

There are currently seven therapies FDA-approved for the treatment of hepatitis B, including interferon (both standard and pegylated), lamivudine, adefovir, entecavir, telbivudine and tenofovir [3]. Factors that will influence treatment choice in women of childbearing age include safety in pregnancy and breastfeeding, efficacy of the agent, intrinsic barrier to resistance and proposed length of therapy. If pregnancy is being contemplated in the near future, it may be prudent to delay therapy until after the child is born [7]. This approach requires a careful assessment of the degree of hepatic activity and fibrosis with either liver biopsy or non-invasive methods. Although it is not recommended in a pregnant woman, interferon can be used in the woman of childbearing age, since therapy with this agent is for a defined period (48 weeks) and often results in clinical remission with HBeAg seroconversion [8]. This is in contrast to the oral antiviral agents that generally require long-term therapy and result in much lower rates of HBeAg seroconversion [3, 7].

For those who require antiviral therapy, it is advisable to discuss the issue of pregnancy before starting treatment. A “planned pregnancy” is preferable and may influence the choice and timing of therapy, or potentially the timing of pregnancy. In addition, given the relative paucity of evidence for most recommendations, decisions about treatment during pregnancy can be made with the luxury of time for consideration of all relevant issues. Treatment with tenofovir is an ideal choice, given its efficacy, high barrier to resistance and safety profile in pregnancy [9, 10]. Lamivudine and telbivudine, two alternate agents considered safe in pregnancy have a substantial incidence of antiviral resistance with prolonged therapy and therefore are no longer first line agents in the non-pregnant patient [3, 7, 10].

## Newly Diagnosed Chronic HBV in Pregnancy

The pregnant woman who is newly diagnosed with HBV early in pregnancy should undergo an assessment of her infection. Decisions about initiating therapy in this setting must include consideration of the risks and benefits for both the mother and the fetus. The risk-benefit equation also depends upon the trimester of the pregnancy. The major determinant of the need for HBV therapy for the mother is the severity of her liver disease (both hepatic activity and fibrosis) [7]. Treatment is generally recommended if the mother is at risk of hepatic decompensation. Most women of childbearing age are likely to have mild disease and thus, treatment can be safely postponed until after delivery. Since many of these women are in the immune tolerant phase of infection (high serum HBV DNA levels with normal ALT and inactive liver biopsy), there is generally no need for therapy and no indication to start therapy during the early stages of pregnancy [3, 7].

## Therapy for Hepatitis B in Early Pregnancy

No antiviral agent has been FDA-approved for use in pregnancy. Thus, when a woman receiving HBV antiviral therapy becomes pregnant, a decision needs to be made whether she should continue therapy for the duration of the pregnancy or if therapy should be withdrawn . As with many decisions during pregnancy, the health of the mother and the fetus must be considered independently. From the perspective of the fetus, the major concern is the risk of exposure to medication during early embryogenesis. From the perspective of the mother, the major issue is whether interruption of antiviral therapy will adversely affect both short and long-term liver disease outcomes. In general, if the mother is known to have significant fibrosis, therapy should be continued since the risk of a flare with withdrawal of therapy could result in decompensation of her liver disease. This effect on the mother’s health could also impact the health of the fetus.

All oral HBV antivirals are inhibitors of either nucleoside or nucleotide polymerases. Although these drugs preferentially target the RNA-dependent DNA polymerase of HBV, they also interfere with replication of mitochondrial DNA and this can result in mitochondrial toxicity leading to the lactic acidosis syndrome [11]. While lactic acidosis syndrome is very uncommon in adults, less is known about the potential ramifications of mitochondrial toxicity in the developing fetus. These may be more diverse, as toxicity may affect organogenesis.

Safety data on HBV antivirals during pregnancy come from two major sources, the antiretroviral pregnancy registry (APR) [10] and also the Development of AntiRetroviral Therapy Study (DART) [12]. The APR is an international, voluntary, prospective exposure registration cohort study of women exposed to antiretroviral therapies, most of whom are HIV-1 mono-infected. As of July 2011, data from 13,507 pregnancies were available. However, this included only 175 women with HBV mono-infection. Table 1 shows the number of cases of early versus late exposure and the rate of birth defects for each of the FDA-approved HBV antivirals. There was no significant difference in the rate of adverse outcomes reported if the initial exposure of any nucleoside or nucleotide drug was in the first trimester (3.0 % and 2.1 %) compared to the second or third trimester (2.5 % and 2.1 %) of pregnancy. In addition, these rates compare favorably to the background 2.72 % rate of defects reported by the CDC birth defect surveillance system. Although these are reassuring data, it is important to look at the experience with each HBV agent. Lamivudine and tenofovir are the two agents with the most in vivo experience in the first trimester and appear to be safe. For telbivudine and entecavir only 8 and 30 pregnancies respectively with exposure in the first trimester are recorded in the registry, with no adverse outcomes reported. Although the APR is extremely useful, it does have limitations including short follow-up and that only defects identified at birth are recorded. Developmental anomalies, such as cardiac or neurological defects identified at a later date, may therefore not be captured.

**Table 1:** Antiretroviral pregnancy registry data

Proportion of defects reported with an exposure to:	Earliest trimester of exposure	
	1st trimester birth defects/live births	2nd/3rd trimester birth defects/live births
Lamivudine	122/3966 (3.1 %)	178/6427 (2.8 %)
Tenofovir	27/1219 (2.2 %)	15/714 (2.1 %)
Telbivudine	0/8	0/9
Adefovir dipivoxil	0/43	0/0
Entecavir	1/30	0/2
Any NRTI	165/5582 (3.0 %)	216/7772 (2.5 %)
Any NtRTI	27/1262 (2.1 %)	15/712 (2.1 %)

The DART study is a 6 year, multi-center, randomized trial of antiretroviral therapy among adults with symptomatic HIV-1 infection or advanced disease/AIDS in Africa. The 3 % rate of congenital anomalies reported in this study also compares favorably to the 2.72 % reported by the CDC birth defect surveillance system [12].

## Continuing, Switching or Discontinuing Therapy?

If the decision is made to continue HBV therapy during pregnancy, the question then becomes whether the drug should be replaced with an agent that has more in vivo experience during pregnancy (i.e. is thought to be “safer”). For example, because of the lack of safety data for entecavir, and because it is an FDA pregnancy class C drug (see Table 2 for FDA pregnancy class definitions [13]), consideration should be given to switching to another agent. Although, the two most commonly used agents in pregnancy are lamivudine and tenofovir, there is a growing experience with telbivudine. Lamivudine is also categorized as a class C agent by the FDA because of reports of toxicity in rabbits with first trimester exposure. However, since it was the first oral agent approved for the treatment of HBV, extensive clinical experience exists. The APR data also suggest that lamivudine is safe despite its pregnancy C classification. However, resistance with short-term use of lamivudine in the third trimester has now been reported [14]. Although there is less clinical experience with tenofovir, it is categorized as a Class B agent by the FDA and has the added benefit of a very high genetic barrier to resistance, with no reported resistance identified to date [13, 15]. Telbivudine, another class B agent [13], has been seldom used for two reasons. Firstly, until the recently published reports that telbivudine reduces perinatal transmission (see section entitled “Evidence for treatment in the third trimester”), there has been minimal in vivo experience in pregnancy with this drug. In addition, it has a low barrier to resistance [16].

**Table 2:** FDA pregnancy categories

Pregnancy Category A	Adequate and well controlled human studies have failed to demonstrate a risk to the fetus in the first trimester of pregnancy (and there is no evidence of risk in later trimesters)
Pregnancy Category B	Animal reproduction studies have failed to demonstrate a risk to the fetus and there are no adequate and well controlled studies in pregnant women or animal studies have shown an adverse effect, but adequate and well-controlled studies in pregnant women have failed to demonstrate a risk to the fetus in any trimester
Pregnancy Category C	Animal reproduction studies have shown an adverse effect on the fetus and there are no adequate and well controlled studies in humans, but potential benefits may warrant use of the drug in pregnant women despite potential risks
Pregnancy Category D	There is positive evidence of human fetal risk based on adverse reaction data from investigational or marketing experience or studies in humans, but potential benefits may warrant use of the drug in pregnant women despite potential risks
Pregnancy Category X	Studies in animals or humans have demonstrated fetal abnormalities and/or there is positive evidence of human fetal risk based on adverse reaction data from investigational or marketing experience, and the risks involved in use of the drug in pregnant women clearly outweigh potential benefits

Rather than switching agents, withdrawal of treatment for the duration of pregnancy may be preferable, especially to the mother who wants to avoid any potential future risk to the fetus. What would be the consequence to the mother of stopping treatment altogether? The natural history of chronic HBV in pregnancy has not been well described. There are limited data to suggest that rarely severe complications of HBV occur late in pregnancy, with reports of liver failure requiring liver transplantation in previously asymptomatic individuals [17]. Data specifically addressing the risk of stopping therapy during pregnancy are anecdotal. Our knowledge of those risks relating to cessation of therapy is derived from early clinical trials in nonpregnant patients, with less advanced fibrosis. In these early studies, therapy was stopped after completion of the trial – even for patients who remained HBeAg(+). Follow-up confirmed that HBV DNA levels rebounded, but rarely did this result in clinically significant flares of hepatitis [18, 19]. In contrast, in those patients with severe fibrosis or cirrhosis at baseline, flares upon treatment withdrawal can result in overt hepatic decompensation [20].

Overall, it appears the risk of an adverse outcome with continuing antiviral therapy during pregnancy is likely very low. However, therapy could be discontinued with close observation of the mother to avoid continued fetal exposure during the first trimester, especially in the patient who does not have advanced fibrosis.

## Rationale for Third Trimester Treatment

The majority of perinatal transmission is thought to occur at delivery, since a combination of passive immunization with hepatitis B immunoglobulin (HBIG) given within 12 h of birth and passive immunization with three doses of the hepatitis B vaccine in the first 6 months of life has resulted in prevention of the majority of infections in this setting. Early seminal studies by Beasley and colleagues showed that HBIG administration could reduce the rate of HBV transmission from >90 % from HBeAg(+) mothers down to ~26 % [21, 22]. When combined with the vaccine, the rates of transmission fell to 3-7 % [23, 24]. In contrast, there have been no convincing data that C-section lowers the rate of perinatal transmission. In a recent retrospective study, 569 infants born to HBeAg-positive mothers found HBV infection in 5.94 % who were delivered vaginally, 8.51 % who were delivered by emergency Caesarean section, and 2.12 % who were delivered by elective Caesarean section [25]. The researchers concluded that elective Caesarean delivery may be the preferred delivery choice to reduce infection risk. However, this has yet to be validated prospectively.

Almost all vaccine and HBIG failures occur in HBeAg(+) women with very high viral loads, generally above 10^8^ copies/mL [26]. A recent report suggested an overall 3 % perinatal transmission rate in viremic mothers despite the use of immunoprophylaxis [27•]. The rate was 7 % in viremic HBeAg positive mothers and 9 % in those mothers who had viral loads >10^8^ copies/mL. Perinatal transmission did not occur in HBeAg-positive mothers with viral loads that were <10^8^ copies/mL. This implies that high viral load, rather than HBeAg status is the main factor for transmission. This has raised the question of whether antiviral therapy before delivery would lower the viral load adequately to prevent transmission.

## Rationale for Third Trimester Treatment

Should pregnant women who are HBsAg positive and highly viremic receive antiviral therapy in the third trimester to prevent perinatal transmission? No consensus on this issue has yet been reached [7]. The principal of treatment late in pregnancy to prevent or reduce the rate of perinatal transmission has been established with other viruses. In HIV-infected mothers antiretroviral therapy (including lamivudine) leads to reduction of mother to child transmission of HIV [28]. Antivirals have also been advocated late in pregnancy to prevent acquisition of neonatal herpes [29].

In a pilot study, 8 women with HBV DNA levels >10^9^ copies/mL were administered lamivudine at 34 weeks of gestation. Babies were vaccinated and received HBIG at birth and only 1 became infected compared to 7 of 25 (28 %) cases of transmission in a matched historical control population [30]. This led to a randomized, double-blind placebo-controlled trial of lamivudine to prevent transmission in highly viremic HBeAg(+) women [31•]. At 1 year of age, 18 % of babies of lamivudine-treated mothers were HBsAg(+) compared to 39 % in the placebo-treated arm. Both groups received vaccination and HBIG. Based on these results, the authors recommended treatment in the third trimester for women with high viral loads. Unfortunately, due to major problems with follow-up, the data are extremely hard to interpret. At 1 year, 13 % of babies in the lamivudine arm and 31 % in the placebo arm had been lost to follow-up. Evaluating only those with complete data, there was a trend but no significant difference in the rate of HBsAg positivity at 1 year (6 % lamivudine vs. 12 % placebo, *p* = 0.37). The study was also very underpowered (power = 53 %), due to slow recruitment. There were no consequences to mother or baby with lamivudine treatment in the study. More recently a meta-analysis to evaluate the efficacy of lamivudine in reducing in utero transmission of HBV has been reported [32•]. A total of ten randomized controlled trials (RCTs) examining 951 HBV-carrier mothers were included [31•, 33–41]. The RCTs evaluated included newborns that received immunoprophylaxis at birth and women were treated with lamivudine from 24–32 weeks of gestation until delivery to 1 month post-delivery. Newborns in the lamivudine group had a 13 % to 24 % significantly lower incidence of intrauterine exposure, indicated by newborn HBsAg (*p* = 0.04) and HBV DNA (*p* < 0.001) positivity. In addition, newborns in the lamivudine treated group had a 1.4-2 % lower perinatal infection rate at 9–12 months indicated by HBsAg (*p* < 0.01) and HBV DNA (*p* < 0.001) seropositivity. This report was limited by the quality of the studies included (most studies were only rated 3 of 5 on the Jadad score [42]) and the heterogeneity of the definition used for high viral load that prompted therapy. A second meta-analysis also reported a decrease in perinatal transmission with the use of lamivudine, but only if the viral load was reduced to <10^6^ copies/mL [43].

In a small study of 31 pregnant women in China, treatment with telbivudine was commenced between 28 and 32 weeks and was continued to 30 days post-partum [44]. Compared with 30 pregnant patients who did not receive therapy and had similar viral loads at baseline and at parturition, the viral loads in the patients treated with telbivudine reduced significantly from 7.38 log10 at baseline to 4.08 log10 prior to parturition (*p* < 0.01). All babies received active and passive immunoprophylaxis. The infection rate was 0 % in those treated with telbivudine and 13.3 % in the untreated controls. Two recent studies have confirmed a reduction in perinatal transmission with telbivudine [45••, 46••]. In the larger of the 2 studies, researchers prospectively enrolled 229 mothers with HBV DNA levels greater than 10 million copies per mL in an open-label trial. From gestational week 20 to week 32, 135 of the women received telbivudine 600 mg/day, while 94 served as controls. All the infants were given HBIG within 12 h postpartum, and received HBV vaccine at 0, 1 and 3 months. Prior to delivery every telbivudine-treated mothers had >3log_10_ reduction in HBV DNA levels, whereas the viral loads in the untreated mothers remained unchanged. Furthermore, 44 (33 %) of the telbivudine-treated mothers and none (0 %) of the untreated controls had undetectable viremia at delivery. Seven months after delivery, the incidence of perinatal transmission was lower in the infants that completed follow-up born to the telbivudine-treated mothers than to the controls (0 % vs 8 %; *p* = 0.002). In the second study, of 88 pregnant women with viral loads of >6 log10 copies/mL, 53 were administered telbivudine starting in the second or third trimester and 35 received no treatment. All infants received standard immunoprophylaxis after birth. At 28 weeks, none of the infants whose mothers received telbivudine had immunoprophylaxis failure, whereas 8.6 % of the infants of control mothers did (*P* = 0.029). In both studies, no serious adverse events were noted in the telbivudine-treated mothers or their infants. Although a large number of patients were enrolled and followed prospectively, the followup of the infants was relatively short. Nevertheless, they contain important data supporting the use of antiviral therapy in the third trimester.

There are no comparable data on efficacy of tenofovir . However, it would be expected that tenofovir would be at least equally effective as lamivudine and telbivudine in reducing perinatal transmission given its potency. This together with its high barrier to resistance makes it an attractive agent to consider in the third trimester.

Overall, additional randomized, multi-center, long-term follow-up studies are needed to better define the utility of antiviral agents in preventing perinatal transmission. In addition, although a high viral load is clearly important, it is not the only factor predisposing to failure of immunoprophylaxis. This is highlighted by a case in which a child developed chronic HBV infection despite suppression of HBV DNA to undetectable levels in the mother with lamivudine therapy throughout gestation and appropriate immunoprophylaxis after birth [47]. Rarely infection may occur in utero, particularly in the setting of threatened pre-term labor with very high maternal viral loads [48]. Furthermore, long-term safety data are lacking and potential risks to the mother include the development of antiviral resistance [14] and flare of hepatitis after treatment withdrawal. In one study 42 % of those who did not receive antiviral therapy during pregnancy experienced a flare in the postpartum period compared to 62 % among those who had been treated and then discontinued therapy at delivery [49]. More recently, it was reported that none of the thirty-eight women who withdrew telbivudine one month post-partum developed severe hepatitis (defined as an ALT > 10 x ULN) [45••].

## Algorithm for Management of HBV in the Pregnant Patient

**Fig. 1:**
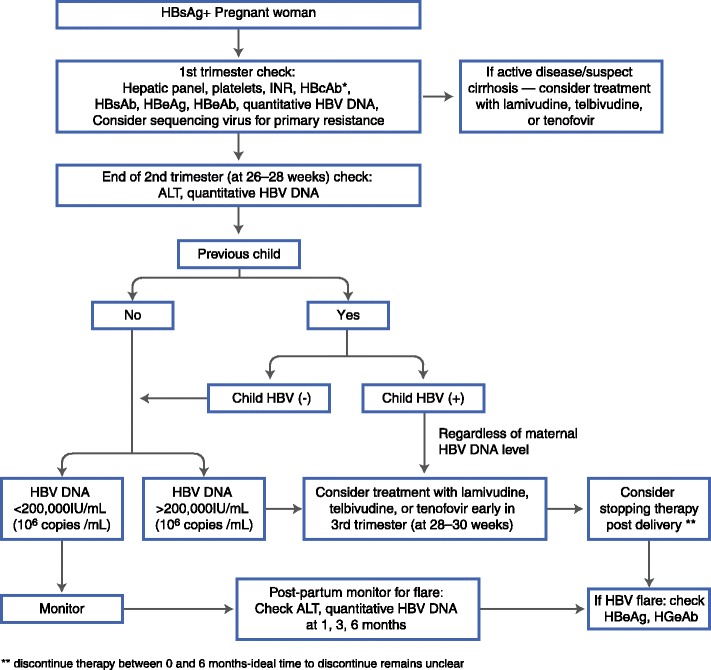
An algorithm for management of HBV in the pregnant patient. Legend: HBsAg = hepatitis B surface antigen, HBcAb = hepatitis B core antibody (total), HBsAb = hepatitis B surface antibody, HBeAg = hepatitis B e antigen, HBeAb = hepatitis B e antibody, ** discontinue therapy between 0 and 6 months-ideal time to discontinue remains unclear

An algorithm for the management and treatment of HBV in pregnancy is proposed in Fig. 1. Routine antepartum care includes testing a woman for the presence or absence of hepatitis B in the first trimester. If she is negative, her child will be vaccinated at birth. The mother does not have to be vaccinated during pregnancy, although it is considered safe and should therefore be administered to those with high risk behavior for acquisition.

For those who test positive early in pregnancy, an assessment of the mother’s HBV status should occur, including a hepatic panel, HBV serologies and platelet count. If the patient has very active HBV (significantly elevated ALT with a high viral load), or if cirrhosis is suspected (low platelet count or suggestive imaging), then therapy should be initiated regardless of trimester. Sequencing of the virus prior to initiating therapy can be considered to aid in deciding which antiviral to use, since primary resistance has been reported [50]. However, if therapy is not warranted (inactive disease with low ALT and low viral load), continued surveillance is suggested since pregnancy can result in a flare of hepatitis B both later in pregnancy and for several months post-partum [17, 49].

It is recommended that all women have their HBV DNA viral load quantified toward the end of the second trimester (at 26–28 weeks gestation) so that a final decision regarding therapy can occur shortly thereafter. This will allow enough time in the third trimester to significantly decrease the viral load after therapy is initiated to decrease perinatal transmission . Women with high viral loads (>200,000 IU/mL or >10^6^ copies/mL) should be considered for therapy early in the third trimester (28–30 weeks), after a thorough discussion of the risks and benefits, since data are too limited to mandate therapy. Once started, therapy is continued for the duration of the pregnancy and can be discontinued post-partum if desired. The decision to discontinue therapy is often influenced by the woman’s desire for subsequent pregnancies. The timing of discontinuation of therapy post-partum remains unclear. In published studies therapy is discontinued anywhere from birth to 1 month post delivery [32•]. In practice, therapy is often continued up to 6 months post-partum. Regardless of when therapy is discontinued, there is a small but real risk of flare and the mother should be monitored closely after withdrawal for at least 6 months. Another factor that may influence the timing of discontinuation of treatment post-partum is the mother’s desire to breastfeed. There are few data regarding the safety of breastfeeding while on antiviral therapy and thus, breastfeeding while on treatment for HBV is not recommended [51, 52].

When deciding on therapy in the third trimester, the perinatal transmission outcome of prior pregnancies must be considered. If previous pregnancies did not result in perinatal transmission, then a viral load of >200,000 IU/mL (10^6^ copies/mL) should be used to determine if therapy should be initiated (similar to the woman who has had no previous children). However, if perinatal transmission did occur with a prior pregnancy, then the risk of perinatal transmission in the current pregnancy is likely higher [7]. In such cases, strong consideration for initiating therapy in the third trimester, regardless of the mother’s viral load at the end of the second trimester is recommended.

## Transmission of HBV Infection in Breastfed Babies

Although early studies did claim that HBV transmission could occur through breast milk, more recent studies have shown that similar rates of acquisition through breast-milk fed and formula-fed babies. In 1975, before the availability of neonatal immunization, the rates of acquisition of HBV were found to be 53 % in breastfed and 60 % in formula-fed babies born to HBsAg positive mothers [53]. These data are limited since the high vertical transmission rates confounded the true rate of acquisition from breast-feeding. After the introduction of immunoprophylaxis, Hill et al. found a similar rate infection rate of breast-fed and formula-fed infants (0 % and 3 %) [54]. A recently published meta-analysis confirms that breastfeeding after proper immunoprophylaxis does not contribute to mother-child transmission of the virus [55]. Thus, current guidelines state that breastfeeding is not contraindicated in HBV-infected mothers not on antiviral therapy whose infants receive immunoprophylaxis [3].

For mothers on antiviral therapy breastfeeding is not recommended. According to prescribing information, it has not been recommended that women breastfeed their infants while taking lamivudine or tenofovir to avoid risking postnatal transmission of HIV-1 infection [51, 52]. Although it is known that lamivudine and tenofovir are both excreted into human breast milk, little is known about the extent of exposure of antiviral agents during breastfeeding. Thus, overall little is known about the safety of breastfeeding in this setting.

## Conclusions

Factors to consider when deciding whether HBV therapy is warranted for either the woman of childbearing age or the one who is pregnant, include extent of existing liver disease, efficacy and safety of existing FDA-approved antiviral agents. Although none of these agents are approved for the use in this setting, there is increasing safety data emerging. With this, there are fewer delays in treating those pregnant women who have clinically active disease. Although it remains unclear if therapy that is initiated in the third trimester in highly viremic mothers reduces perinatal transmission, published reports suggesting a benefit are emerging. Additional randomized, multicenter, long-term follow-up studies are needed.
